# Effects of obesity on clinical outcomes in diminished ovarian reserve patients undergoing intracytoplasmic sperm injection cycles

**DOI:** 10.1097/MD.0000000000038942

**Published:** 2024-07-12

**Authors:** Belgin Devranoğlu, Müşerref Banu Yilmaz, Gamze Peker, Özlen Emekçi Özay, Ali Cenk Özay, Ali İrfan Güzel

**Affiliations:** a Health Sciences University, Zeynep Kamil Women and Children’s Diseases Training and Research Hospital, İstanbul, Turkey; b Department of Obstetrics and Gynecology, Ümraniye Training and Research Hospital, İstanbul, Turkey; c Department of Obstetrics and Gynecology, Cyprus International University, Nicosia, Cyprus; d Department of Obstetrics and Gynecology, Sanko University, Gaziantep, Turkey.

**Keywords:** clinical pregnancy rate, diminished ovarian reserve, obesity

## Abstract

The aim of this study is to evaluate the effects of obesity on clinical outcomes in diminished ovarian reserve (DOR) patients undergoing intracytoplasmic sperm injection cycles. In this retrospective observational cross-sectional study, women admitted to current clinic with DOR undergoing intracytoplasmic sperm injection were divided into 2 groups according to the obesity. Patient age, body mass index, anti-mullerian hormone, baseline follicle stimulating hormone and baseline estradiol levels, antral follicle count, total gonadotropin dose, day of stimulation, number of mature (MII) oocytes, and clinical pregnancy were evaluated. There were no statistically significant differences between groups in terms of age, anti-mullerian hormone, baseline follicle stimulating hormone, baseline estradiol levels, antral follicle count, and clinical pregnancy (*P* > .05). Total gonadotropin dose, the days of ovarian stimulation were higher and number of MII oocyte were less in the obese group (*P* < .05). Logistic regression analyses also revealed that the days of ovarian stimulation and number of MII oocyte were significant factors in the study group. ROC curve analysis showed obesity is a negatively affecting factor in DOR patients. Obesity causes more gonadotropin dose longer days of stimulation, and less number of MII oocyte. However clinical pregnancy rate is not negatively affected by obesity according to the current study.

## 1. Introduction

The purpose of intracytoplasmic sperm injection/in vitro fertilization (ICSI/IVF) procedure is to achieve the highest pregnancy rates with the lowest complication rates.^[1]^ It is known that more than a third of women of reproductive age suffer from obesity. Obesity is a dangerous risk factor for infertility.^[2–5]^ In women undergoing ICSI, obesity is known to result in a higher dose of gonadotropins per cycle, a lower number of MII oocytes, poorer oocyte quality, poorer embryo quality, and a higher cycle cancelation rate.^[6]^ In addition, obese women have lower implantation rates, clinical pregnancies, and live births after ICSI with their own eggs.^[7,8]^

Cytokines take part in different roles in ovaries. Folliculogenesis is dependent on activity of cytokines, and knotted relationship of obesity and inflammation could adversely influence folliculogenesis and other reproductive processes at the cellular level.^[9,10]^ Furthermore, hormonal discrepancies, together with a mixture of disorders related to insulin, decreased levels of sex hormone binding proteins, and increased androgen levels, add to this phenomenon.^[11]^

In the current study, we investigated the effects of obesity on clinical outcomes in diminished ovarian reserve (DOR) patients undergoing ICSI cycles.

## 2. Materials and methods

### 2.1. Study design and participants

This retrospective observational cross-sectional study was designed in the Department of Obstetrics and Gynecology, Division of Infertility and Gynecological Endocrinology, at Zeynep Kamil Women’s Health Education and Research Hospital. This is a tertiary referral research hospital located in the biggest city of Turkey.

Diminished ovarian reserve was defined by the following criteria: a basal follicle stimulating hormone (FSH) level >10 IU/L, an antral follicle count <6 or a previous poor ovarian response. The presence of at least one of these criteria was required to make the diagnosis of poor ovarian reserve.

### 2.2. Ethical considerations

The study was also approved by the research ethics committee of Zeynep Kamil Women’s Health Education and Research Hospital (İstanbul, Turkey) (Ethical approval date: March 6, 2024, No: 34).

### 2.3. Data collection

Women who had infertility (primary or secondary) with DOR for at least 1 year were included to the study. A total of 833 IVF/ICSI procedures which were performed between June 2019 and June 2022 were enrolled into the study. Data were collected from the hospital record and patient’s files.

Factors recorded were; age, body mass index (BMI), cycle day 3 serum FSH, and estradiol (E2) levels, anti-mullerian hormone levels, antral follicle count, total gonadotropin dose and day of ovarian stimulation, number of MII oocytes, and clinical pregnancy rates. The patients were divided into 2 groups according to BMI; group 1 (n: 564; BMI < 30 kg/m^2^) versus group 2 (obese group) (n: 269; BMI ≥ 30 kg/m^2^).

### 2.4. Stimulation regimen and ICSI/IVF procedure

For all of the cases standardized gonadotropin-releasing hormone antagonist protocol was used. Before ovarian stimulation, all patients had transvaginal ultrasound examination on 2nd or 3rd day of menstruation. On that day recombinant FSH (Gonal-F, Serono, Switzerland) with dose between 150 and 300 IU daily was started according to patients’ characteristics. Transvaginal ultrasound examination was performed at the 5th day of treatment and repeated once in every 2 or 3 days for follicle growth and endometrial thickness inspection. Besides, transvaginal ultrasonography is performed and serum E2 levels were monitored. A daily injection of 0.25 mg of the antagonist (Cetrorelix, Merck-Serono, Geneva, Switzerland) was added when the diameter of the leading follicle reached ≥12 mm until day of trigger. In poor responder patients, when a dominant follicle reached 17 to 18 mm altogether with E2 levels that are >500 pg/mL, ovulation is triggered by 10,000 units of human chorionic gonadotropin. After 34 to 36 hours, oocyte pick up is performed. According to semen quality on the day of oocyte retrieval, the oocytes were subjected to ICSI.

### 2.5. Embryo transfer

Embryo transfer was determined based on the American Society for Reproductive Medicine guidelines^[7]^ and was performed using a Wallace catheter (Edwards‐Wallace catheter; Marlow Technologies, Willoughby, OH) by the same clinical team. Gametes were prepped in GMOPS medium (Vitrolife, Frolunda, Sweden) plus 0.3% human serum albumin. After confirming fertilization, embryos were cultured in GMOPS + HSA until day 3. Embryos not selected for transfer on day 3 were placed in G-2 (Vitrolife, Frolunda, Sweden) plus HSA for extended culture.

### 2.6. Luteal phase support and clinical pregnancy determination

All patients received luteal phase support with 90 mg/day vaginal progesterone gel (Crinone 8%, Merk Serono, Germany) starting on the day of oocyte retrieval. Clinical pregnancy was defined as the presence of gestational sac containing fetal hearts on ultrasound scan. Clinical pregnancy rates and cancelation rates were calculated per the number of IVF treatment cycles.

### 2.7. Statistical analyses

Statistical analyses were undertaken with Statistical Package for Social Sciences for Windows 17.0 (SPSS Inc., Chicago, IL). The Kolmogorov‐Smirnov test was used to establish whether or not numeric data exhibited a normal distribution, and the percentage was expressed as mean ± standard deviation. Data demonstrating a normal distribution were analyzed with the Student *t* test. Logistic regression analysis was used to find out the independent risk factors and ROC curve was used to define the discriminative power of these parameters. For the results thusly obtained, 95% confidence interval and *P* < .05 were regarded as preconditions for statistical significance.

## 3. Results

The demographic and clinical parameters of the cases are depicted in Table 1. There was no statistically significant difference between the groups in terms of age, serum anti-mullerian hormone levels, baseline FSH and E2 levels, and antral follicle count (*P* > .05).

**Table 1:** Comparison of demographic and clinical features of patients in obese and nonobese group.

	Non-obese group (n = 564)	Obese group (n = 269)	*P* value
Age (years)	35.9 ± 4.88	36.0 ± 4.74	.912
AMH (ng/mL)	0.47 ± 0.41	0.53 ± 2.09	.478
Baseline FSH (mIU/mL)	12.2 ± 7.87	11.3 ± 7.55	.832
Baseline estradiol (pg/mL)	57.6 ± 7.55	61.7 ± 7.88	.354
AFC	3.4 ± 1.89	3.7 ± 1.98	.069
Total gonadotropin doze (IU)	3636.7 ± 1192.33	3819.7 ± 1273.34	**.043**
Day of stimulation	9.1 ± 2.33	9.5 ± 2.26	**.023**
No of MII oocytes	1.7 ± 1.77	1.4 ± 1.60	**.032**
Clinical pregnancy	13	35	.427

AFC = antral follicle count, AMH = anti-mullerian hormone, FSH = follicle stimulating hormone.

Total gonadotropin dose, days of stimulation and number of MII oocytes were statistically significant between the groups (*P* < .05). The clinical pregnancy rate was not different between the groups (*P* > .05).

According to the correlation analysis that performed in obese group, clinical outcomes including; higher total gonadotropin dose, longer days of stimulation, and lower number of MII oocytes were negatively affected in the obese group (Table 2).

**Table 2:** The correlation analysis of clinical outcomes among obese group.

	β	SE	Wald	Odds ratio	*P* value
Total gonadotropin dose	0.043	0.310	6.162	1.257	**.026**
Days of stimulation	0.544	0.458	4.155	1.228	**.031**
Number of MII oocytes	0.322	0.022	5.025	1.633	**.048**

**Figure 1.**
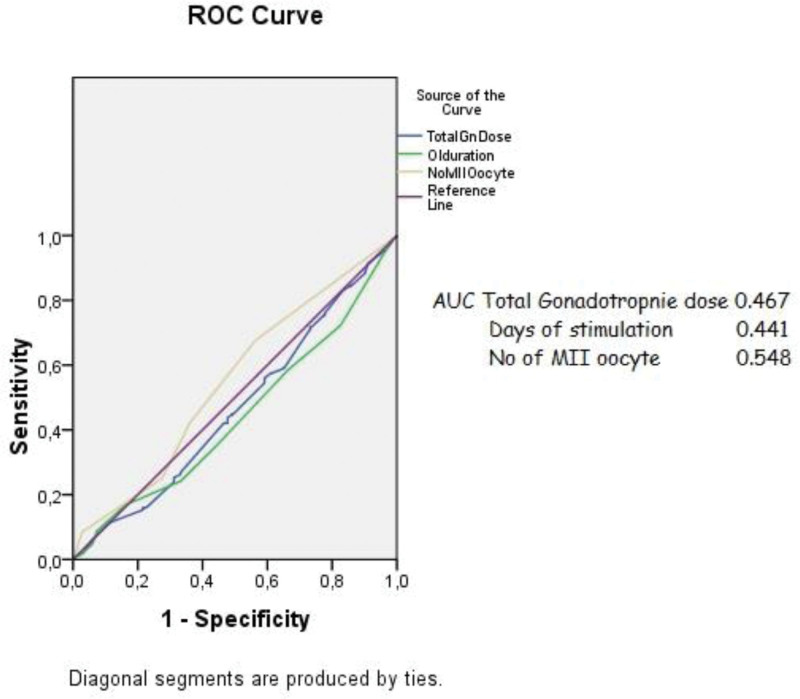
ROC curve analysis of clinical outcomes in obese group.

ROC curve analysis (Fig. 1) demonstrated the area under curve for total gonadotropin dose, days of the stimulation and number of MII oocytes depicted the area under curve, cut off value, and sensitivity and specificity of these variables.

## 4. Discussion

The present study aimed to investigate the effects of obesity on clinical outcomes in patients with DOR undergoing ICSI cycles. Our findings suggest that while obesity may increase gonadotropin requirements and duration of ovarian stimulation, it does not appear to have a significant negative effect on clinical pregnancy rates in this population. Also number of MII oocytes were negatively affected in obese group.

Obesity is known to be associated with alterations in reproductive function, including disruptions in menstrual cycles, ovulatory dysfunction, and decreased fertility.^[12]^ These effects are thought to be mediated by various mechanisms, such as alterations in hormone levels, insulin resistance, and inflammation.^[13]^ In the context of assisted reproductive technologies, obesity has been linked to poorer outcomes, including lower pregnancy rates, increased miscarriage rates, and higher rates of pregnancy complications.^[7]^ However, the specific impact of obesity on outcomes in patients with DOR undergoing ICSI has been less well-studied.

Our study found that obese patients with DOR required higher total gonadotropin doses and longer durations of ovarian stimulation compared to nonobese patients. These findings are consistent with previous studies that have reported associations between obesity and suboptimal response to ovarian stimulation in both in vitro fertilization and ICSI cycles.^[14]^ One possible explanation for this observation is that obesity is associated with alterations in ovarian function, including decreased sensitivity to gonadotropins and impaired folliculogenesis.^[15]^ Additionally, obesity is often accompanied by insulin resistance and hyperinsulinemia, which may negatively impact ovarian function and response to ovarian stimulation.^[16]^

Interestingly, despite the higher gonadotropin doses and longer duration of ovarian stimulation observed in obese patients, clinical pregnancy rates were similar between obese and nonobese patients in our study. This finding contrasts with some previous studies that have reported lower pregnancy rates in obese patients undergoing assisted reproductive technologies.^[17]^ One possible explanation for this discrepancy is that our study focused specifically on patients with DOR, who may already have compromised ovarian function regardless of obesity status. In this population, factors other than obesity, such as age and ovarian reserve, may play a more dominant role in determining treatment outcomes. Indeed, our study found no significant differences in age or baseline ovarian reserve parameters between obese and nonobese patients, suggesting that these factors were well-balanced between groups.

The lack of a significant difference in clinical pregnancy rates between obese and nonobese patients may also be related to advances in assisted reproductive techniques and protocols. For example, the use of personalized ovarian stimulation protocols, including gonadotropin-releasing hormone antagonist protocols and individualized dosing regimens, may help to optimize treatment outcomes in obese patients.^[18]^ Additionally, the use of techniques such as preimplantation genetic testing for aneuploidy may help to identify euploid embryos for transfer, thereby improving pregnancy rates in this population.^[19]^

It is important to note some limitations of our study. Firstly, as a retrospective study, our findings are subject to potential biases inherent in the study design. Additionally, the relatively small sample size may have limited our ability to detect small differences in clinical outcomes between groups. BMI is considered as gold standard by which obesity is measured and defined.^[20]^ Since increased BMI could affect IVF outcomes in different ways, positively^[21]^ or adversely^[22]^ according to cause of infertility, we acknowledge the lack of BMI evaluation as another limitation of the study.

Future studies with larger sample sizes and prospective designs are needed to confirm our findings and further elucidate the impact of obesity on outcomes in patients with DOR undergoing assisted reproductive technologies.

## 5. Conclusion

Our study suggests that while obesity may impact certain aspects of the treatment process in patients with DOR undergoing ICSI cycles, it does not appear to have a significant negative effect on clinical pregnancy rates. These findings highlight the importance of personalized treatment approaches and the need for further research to optimize outcomes in obese patients undergoing assisted reproductive techniques.

## Author contributions

**Conceptualization:** Özlen Emekçi Özay.

**Data curation:** Belgin Devranoğlu, Müşerref Banu Yilmaz, Gamze Peker, Özlen Emekçi Özay, Ali Cenk Özay, Ali İrfan Güzel.

**Formal analysis:** Belgin Devranoğlu, Müşerref Banu Yilmaz , Gamze Peker, Ali Cenk Özay.

**Investigation:** Ali İrfan Güzel.

**Methodology:** Ali İrfan Güzel.

**Writing – original draft:** Ali İrfan Güzel.

**Writing – review & editing:** Ali İrfan Güzel, Ali Cenk Özay.
